# A qualitative analysis of clinician perspectives on community health worker integration at epilepsy centers

**DOI:** 10.3389/fneur.2025.1560077

**Published:** 2025-04-09

**Authors:** Elaine T. Kiriakopoulos, Barbara Jobst, Anna Murray, Carly Sykes, Suzanne Lenz, Trina Dawson, Wren M. Kaden, Felicia Chu

**Affiliations:** 1 Geisel School of Medicine, Dartmouth College, Hanover, NH, United States; 2 HOBSCOTCH Institute, Dartmouth Hitchcock Medical Center, Dartmouth Health, Lebanon, NH, United States; 3 University of Massachusetts Chan Medical School, Worcester, MA, United States

**Keywords:** epilepsy, social determinants of health, community health workers, health equity, care coordination, interdisciplinary teams

## Abstract

**Introduction:**

Substantial evidence supports community health worker (CHW) interventions lead to improved health outcomes, but health professionals’ perceptions of CHW roles and effectiveness may slow integration into care delivery systems. Research on CHWs as members of specialty care teams in clinical settings, especially in the epilepsy field, is limited.

**Methods:**

We conducted semi-structured interviews between April and October 2022 to explore perceptions of multidisciplinary clinicians (*n* = 12; physicians, nurses, social workers) based at five New England epilepsy centers about the potential for successful CHW integration on specialist epilepsy center care teams. Transcripts were analyzed using a mixed inductive and deductive thematic approach.

**Results:**

Themes that emerged included: (1) limited epilepsy clinician awareness of the CHW role and minimal experience working with a CHW; (2) limited clinician knowledge of how to recruit CHWs; (3) preference for CHW roles and responsibilities in epilepsy centers being focused on social determinants of health (4) clinician uncertainty around scope of CHW training and supervision; (5) unknown funding mechanisms for CHW integration in epilepsy centers; (6) knowledge of care gaps in addressing social determinants of health needs by epilepsy centers; (7) openness by epilepsy center care team members to CHW integration; and (8) environment and collaborative culture at epilepsy centers.

**Discussion:**

Despite limited knowledge and experience regarding CHW roles and mechanisms for recruiting and sustaining CHW positions, the multidisciplinary clinicians interviewed valued the potential benefits of CHWs in an epilepsy center and endorsed a need to better address patients’ unmet social determinants of health needs.

## 1 Introduction

Community health workers (CHW) are traditionally positioned in community-based settings as valuable frontline health professionals who act as liaisons between healthcare systems, social services, and the communities they serve (1). In the United States, CHW interventions have demonstrated predominantly positive effects on health outcomes, including in populations who often face significant disparities related to health care access and quality (2, 3). As a result, CHWs are increasingly perceived as important members of community service and primary health care delivery teams (4–9). Epilepsy presents unique medical and social challenges that often make disease management complex, and the consequences of epilepsy are exacerbated by the substantial disparities that exist in accessing care (10). Many factors contribute to whether an individual with epilepsy receives care, including socio-economic status, education, insurance coverage, and transportation (11–13). CHWs could be engaged to facilitate access to community-based resources to overcome such barriers to care that people with epilepsy often face. No widely accepted evidence-based approaches for integrating CHWs into epilepsy health care settings have been developed.

Growing research on the role of CHWs in managing chronic conditions suggests that CHWs could support people with epilepsy with their needs around improved care coordination, disease management, and with addressing unmet social needs that impede health (2, 14–21). Pilot data demonstrated feasibility for training and integrating CHWs into epilepsy center teams resulting in high provider and patient satisfaction, and the potential for cost savings is supported in the literature (22, 23). Despite this promising evidence and growing interest in addressing social determinants of health (SDOH) gaps among epilepsy patients, there are no models for integrating CHWs in epilepsy care.

The purpose of this study was to examine epilepsy center clinicians’ perceptions regarding factors that would facilitate integration of CHWs into epilepsy care teams, inclusive of issues related to recruitment, training, supervision, roles, and responsibilities. This inquiry will augment information in support of CHW program design and implementation strategies at epilepsy centers. All research procedures were approved by the University of Massachusetts and the Dartmouth Health Institutional Review Boards.

## 2 Materials and methods

We used reflexive thematic analysis as practiced by Braun and Clarke that facilitates author consensus (24–28).^1^ We used the Standards for Reporting Qualitative Research guidelines to guide our qualitative methodology (29).

### 2.1 Interview participants

Key informants were purposively selected based on (1) their primary clinical roles being centered at an accredited epilepsy center and (2) having leadership experience or ongoing influence within their epilepsy center care teams or clinical practice. Participating key informants (*n* = 12) represented diverse clinical roles inclusive of epileptologists, epilepsy specialist nurses, and clinical social workers based at epilepsy centers. The cohort included clinicians serving patients from urban, suburban, and rural regions across New England.

### 2.2 Semi-structured interview

A semi-structured interview guide (Supplementary Materials: Appendix A) was developed by two researchers (EK, FC) and addressed eight topics investigated initially by literature review and quantitative digital survey. Topic areas explored in the interviews included: (1) Prior knowledge or experience with CHWs; (2) Recruitment and selection of CHWs; (3) CHW roles and responsibilities; (4) CHW training and supervision; (5) CHW funding mechanisms; (6) Current epilepsy care gaps; (7) Clinical care team expectations and trust; and (8) Epilepsy center environment and culture of collaboration. These select topics served as parent codes for the current qualitative analysis. The initial draft of the semi-structured interview guide was reviewed by a multidisciplinary expert panel with relevant experience and the feedback received resulted in a reordering of the main question categories and the addition of a clarifying question requesting specific examples was added. Interview questions were then pilot tested with a public health researcher and with epilepsy clinical providers who had characteristics similar to eligible key informants. This allowed for refining the precise wording of interview questions and determining the interview length. A complete list of codes can be found in Supplementary Materials: Appendix B, including codes that were derived from the quantitative survey but were not applicable to the qualitative interviews.

### 2.3 Data collection

Interviews were conducted (April to October 2022) one to one via WebEx (audio only with automated transcription) by trained experienced interviewers (EK, FC). See Supplementary Materials: Appendix A for the clinician interview guide. Informed consent was obtained. We recorded all interviews, then used WebEx software to transcribe them verbatim. Interviews lasted between 45 and 60 min. An iterative approach to collecting, analyzing and reviewing data allowed for the research team to evaluate saturation. Thematic saturation was reached at the culmination of 12 interviews, determined by no new codes or themes under existing codes identified during iterative coding analysis; and by consensus between the interviewers (EK, FC) that redundancy of perceptions and observations shared by the key informants was noted during data collection.

### 2.4 Data analysis

Each transcript was reviewed by the study coordinator and two researchers (EK, FC) to check for potential errors by the transcribing WebEx software and to familiarize the researchers with the data collected. We used a mixed inductive and deductive approach to code and analyze the data. First, an initial round of open coding was completed on three transcripts by two researchers (FC, EK), utilizing eight topic areas as *a priori* parent codes to group the interview responses by topic area. Transcripts were then coded independently by four researchers (AM, FC, CS, EK) into topic areas using a deductive, iterative process. Multiple coders, including individuals not involved in the interview process, minimizes research subjectivity. Child codes were created along with marking meaningful text within each of the parent code categories to further group responses by topic categories. The team compared the lists of codes developed iteratively to inform and refine the development of a final codebook. To achieve this the research team met regularly to compare notes on analysis of transcripts examining for differences or discrepancies, and to reach a consensus on the definitions of codes and coding of text units. The final codes were reviewed and confirmed during meetings between all four coders (EK, AM, FC, CS). Through direct discussion and comparison, a shared understanding of coding practices served to further build consensus and strengthen credibility of analysis. Two subsequent rounds of thematic analysis coding were then completed by three researchers (AM, FC, CS) to identify common themes. Intra- and inter-coder reliability were evaluated, and themes were refined with all team members. Select supporting phrases, representing themes by diverse clinician perspectives, were included. Each thematic category is described at length.

### 2.5 Researcher characteristics and quality

We built an interprofessional research team including three neurologists, one nurse, two community health workers, and two public health researchers with qualitative research training. Additionally, we discussed findings with relevant CHW, epilepsy, and public health stakeholders, including community members, partners and people affected by epilepsy center care practices for interpretation. We present our findings using thick description [i.e., context is incorporated in description of observations (30)] and quotations from key informant interviews.

## 3 Results

We completed semi-structured interviews with 12 multidisciplinary clinicians from five academic Level 3 and 4 epilepsy center teams (given long term objective of integrating CHWs in centers where people with epilepsy receive comprehensive specialist care) in New England caring for patients in Massachusetts, New Hampshire, Vermont, Maine, and Connecticut. Interview participants had diverse clinical roles inclusive of physicians (*n* = 8; junior faculty *n* = 2, mid-career *n* = 3, epilepsy chief *n* = 3), nurses (*n* = 2), and clinical social workers (*n* = 2).

### 3.1 Thematic analysis

**Figure 1:**
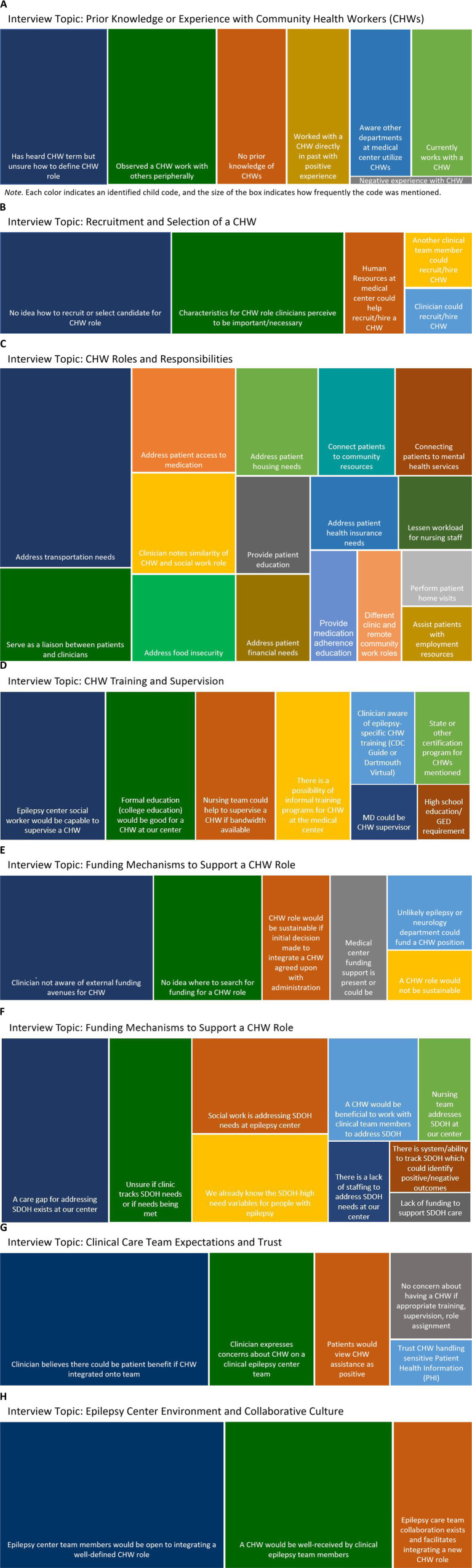
**(A)** Theme: There is limited clinician awareness of the CHW role and minimal firsthand experience working with a CHW across disciplines. **(B)** Theme: There is limited clinician knowledge on how to best recruit and select a CHW. **(C)** Theme: CHW epilepsy clinic roles and responsibilities should be centered around social determinants of health with an emphasis on transportation, medication access, and a service role of clinician-patient liaison. **(D)** Theme: Clinician uncertainty around type and specificity of CHW training and supervision is high. **(E)** Theme: Funding mechanisms to support and sustain a CHW role at the epilepsy center are not known or readily available, presenting a significant barrier to CHW integration. **(F)** Theme: SDOH care gaps are present at epilepsy centers and require a systematic approach with dedicated staff time and systems to screen, identify, address and track. **(G)** Theme: Clinicians support well-structured CHW integration which has potential to benefit epilepsy patients. **(H)** Theme: Clinicians endorse the idea that an epilepsy-trained CHW could experience a sense of belonging on an epilepsy center care team and be acknowledged as a contributor so long as the CHW role is well-defined and supervision is in place.

Themes emerging from semi-structured key informant interviews included: (1) limited epilepsy clinician awareness of the CHW role and minimal experience working with a CHW; (2) limited clinician knowledge on how to recruit CHW; (3) preference for CHW epilepsy clinic roles and responsibilities being focused on SDOH; (4) clinician uncertainty around scope of CHW training and supervision; (5) unknown funding mechanisms for CHW integration in epilepsy centers; (6) knowledge of care gaps in addressing SDOH needs by epilepsy centers; (7) openness to CHW integration on an epilepsy center care team; and (8) environment and collaborative culture at epilepsy centers. Each theme is visually displayed using tree maps, each of which conveys the relative weight (or frequency) of each child code by topic discussed during interviews. Codes represented by larger areas were heard more often than codes in smaller areas; each mention of the child code by an interviewee was a count of 1 (Figure 1).

### 3.2 Representative quotes

Results in the following section highlight supporting representative quotes by theme.

#### 3.2.1 Prior knowledge or experience with CHWs

Respondents described limited understanding of the CHW role designation (Figure 1A), with some respondents having little to no prior knowledge of the CHW profession. Across disciplines there was very little firsthand experience described of directly engaging with a CHW in the care of patients; respondents were much more likely to indicate having observed a CHW working with others peripherally or some awareness of other care teams at the clinical center who utilize CHWs. The majority of past interactions with a CHW were viewed positively.

*“I honestly have never worked with a community health worker and I’m not sure I knew they existed.”* (Physician)

*“I do have some knowledge about community health workers, because I’ve worked sort of tangentially with them in the past, because in my previous role I was a nurse for an agency that served developmentally disabled population*.” (Nurse)

#### 3.2.2 Recruitment and selection of CHWs

Respondents predominantly described being unsure how they would go about recruiting a CHW for their epilepsy center (Figure 1B), possibly in part due to some having unfamiliarity with the CHW role. Some respondents shared a general awareness of CHWs having roles at their medical centers. Clinicians expressed uncertainty around the selection criteria to consider when hiring a CHW, and few felt that they or a member of their team could perform this task. A recruitment and hiring process involving the Human Resources department at their clinical center was viewed as more feasible. Clinicians frequently identified desirable CHW candidate characteristics, including having some prior experience working with patients, at least some college education, knowledge of community resources, “good judgment,” and life experiences a patient could identify with.

*“I guess I would want someone who has some prior patient experience, some interest in working with patients, I do not know if that would entail you know, psychology training, social work training.”* (Physician)

*“Someone within the community, so someone with [community] knowledge, someone probably culturally competent.”* (Nurse)

#### 3.2.3 CHW roles and responsibilities

Across disciplines, clinicians identified a number of responsibilities that a CHW would be well suited to address (Figure 1C). The most-frequently cited role by far was to address patient transportation needs, although medication access, food insecurity, and housing insecurity were also commonly mentioned. Interviewees also shared that it would be helpful to have a CHW on an epilepsy center team serve as a communication liaison between the provider and patient during and beyond clinic appointments to follow up on medication access and adherence, transportation to the next clinic visit, and connecting the patient with mental health services or medical testing. Several respondents noted the similarity and overlap between social work and CHW roles and the possibility for the roles to work in synergy. Several respondants indicated this potential synergy could reduce nursing staff workload.

*“Helping with housing, helping with disability, helping with employment, helping with medication adherence.”* (Physician)

*“I can imagine that the role might also involve educating, not just patients, but caregivers and other members in the community about the disorder of epilepsy.”* (Physician)

*“…I mean, you know, helping them obtain access to resources, such as, housing, I guess, you know, food, just because I know that’s, you know, I know a lot of my patients have a lot issues with finances and, like, how to get the bills paid on time and you know, access to, other providers.”* (Nurse)

*“…Given the fact that epilepsy patients traditionally have issues with transportation; it would [also] be great to have a community health worker, who would actually be able to literally go into the patient’s community and meet them more where they are at their home, which we do not really have that luxury to do at this moment.”* (Nurse)

#### 3.2.4 CHW training and supervision

Respondents revealed they had little to no awareness of how CHWs were trained and of whether there are certification or licensing requirements for the role (Figure 1D); again, this is likely related to general unawareness of CHWs, and their potentially role in the epilepsy clinic specifically. Respondents mostly preferred at least some college education among candidates for an epilepsy center CHW role, although a few indicated a high school diploma or GED may be sufficient. Only a few interviewees were aware of existing accredited epilepsy-specific training for CHWs. The majority expressed that social worker or nursing supervision would work best for CHW but recognized these staff have limited bandwidth that would allow adding additional duties to their workload.

*“And so as long as we were clear what the tasks are. And then the training that is provided is the training around those tasks then I think we should be good.”*(Physician)

*“I’d like to think they, you know, would know what epilepsy is and you know how the treatment plan works, meaning, you know, there’s no algorithm for epilepsy.”*(Nurse)

*“I feel like the physicians might be a little overwhelmed if they had to do that [supervision]… we have nurse navigators that might be able to do that [supervision] as well.”* (Nurse)

*“The important things are being on a team with supervision and also having clear boundaries, establishing boundaries.”* (Social Worker)

#### 3.2.5 Funding mechanisms to support a CHW role

No respondent was aware of a mechanism to fund a CHW (Figure 1E), although a few felt it might be possible through their medical center. Some suggested that if value could be demonstrated and administration committed to supporting a CHW for the epilepsy center, then a CHW integration program had potential for becoming sustainable. Others shared a less optimistic outlook in the current healthcare environment with budget constraints prevailing, indicating that it was unlikely the epilepsy or neurology department at their center would be willing or able to support a CHW. Many respondents indicated they simply did not know where to search for funding for a CHW role.

“*I think what you’d need to do is get some departmental buy-in and show we have this burden of administrative tasks that we could be using to be more clinically productive. And with investment in community health workers, I could be seeing more clinic patients in clinic or seeing patients in clinic more quickly, or things like that. Because I actually do think it could increase clinic efficiency.”* (Physician)

*“I think the thing I would emphasize is like, all things I’d see the biggest barrier just being financial resources to acquire one and convincing a department that it’s worth it.”* (Physician)

#### 3.2.6 Identifying current epilepsy center care gaps

Interviewees across disciplines acknowledged care gaps related to addressing patient SDOH needs (Figure 1F) and stated that they often relied on social workers and nurses to try to meet needs as well as possible. Lack of staffing and funding were reasons provided for the care gap despite systems in place to track SDOH at some centers. Clinicians shared that the SDOH needs of people with epilepsy are well known and even high need variables such as transportation could be challenging to address in a clinic visit. A high number of clinicians were unsure SDOH were being tracked in their patients, or if those needs were being met. Again, many respondents noted that CHWs could be potentially beneficial in addressing these needs.

*“With the patient population we have, I feel like there’s plenty of unmet needs.”* (Social Worker)

*“And I think the larger gap is that there are people who are not reaching our care or being able to sustain their care for with us in the 1st place and that’s probably the bigger gap is that people come and we have plans for them and then, because of factors outside of their control they are not able to keep coming to see us.”* (Physician)

*“Oh… I mean, if you look at the housing, if you look at food, if you look at access to housing, rides to get to your appointment, money to pay for your food. Mm. Hmm. Insurance issues, everything, you know, even you know, the emotional support which is there. You know, hooking people up with elder services, you know, the grants, education.”* (Social Worker)

#### 3.2.7 Clinical care team expectations and trust

The majority of clinician perspectives favored CHW integration, citing likely patient benefit and that patients would probably view the CHW’s assistance as helpful (Figure 1G). Several interviewees mentioned that handing sensitive protective health information would be an important part of the CHW’s training, but that they would trust a CHW to manage this information securely. Concerns focused on the CHW as a non-clinician requiring appropriate guidelines for training, supervision, and a clear delineation of CHW responsibilities.

*“I would say I’m quite confident. I mean, honestly, as long as they are, I do not want to use this expression, but like, staying in their lane, and they are not talking to patients to, like, change their medications and then things like that [then] I’m very confident in their ability to help.”* (Physician)

*“I mean, half the time, what you need is someone who has the time and patience to talk to the patient, find out what’s really going on at home find out what the barriers [to] medication adherence are find out what the social situation is. And that does not necessarily require a medical degree that a lot of that is just people skills and just enjoying working with people and detective work.”* (Physician)

*“…especially if they go into the home, you know, they would definitely be able to bring back a different perspective of the patient, and I think that just having, like I said, almost being non-medical might be to an advantage because they might be perceived different by the patient a little bit more.”* (Nurse)

#### 3.2.8 Epilepsy center environment and collaborative culture

Respondents had a high degree of interest regarding integrating a CHW on an epilepsy care team (Figure 1H). Interviewees felt if concerns around training, role definition, and supervision were met, then a CHW could function as a contributing member of a collaborative interdisciplinary team caring for people with epilepsy, viewed positively by team members. Existing epilepsy care team collaboration was noted as a facilitator of CHW integration by some.

*“I mean, I think, you know, I think they would add so much to our team. You know, providing, you know, just community education and social support, and just kind of be a liaison…”* (Nurse)

*“I think, you know, the biggest thing is just to make sure that they [CHW], especially if they are going to be not spending as much time in the clinic as the rest of the staff, that they feel very much part of the epilepsy team, and that they are involved in all the meetings.”* (Nurse)

## 4 Discussion

Despite limited experience with CHWs, providers indicated that epilepsy care center teams would likely experience benefit with CHW integration to improve patient-centered care and promote health equity. As healthcare systems increasingly prioritize patient-centered, team-based care models (31–34), the role of CHWs is likely to continue to evolve and expand as an integral part of interdisciplinary care teams in specialty practice settings. Our data demonstrates that epilepsy center clinicians recognize that patient SDOH care gaps need more attention and that they view a potential workforce to help identify and address needs as worthy of consideration.

We found some perceived barriers to CHW integration, including external funding, internal department or medical center financial support, and role clarification. Notably, there was an imperative to clearly delineate roles and expectations for a CHW on an epilepsy center team to preclude any influence over clinical care decision making. These finding parallels prior research supporting clarity among team members leading to successful CHW integration (35, 36). Findings also highlight the need for a concerted effort to integrate a CHW onto the team in a purposeful way that would allow the CHW to feel they were contributing meaningfully to patient care. The low level of awareness among epilepsy providers regarding CHWs, in general, underscores the need for an onboarding schema that informs clinical team members and clinic support staff of the evidence of the benefit of CHWs in chronic disease management and facilitates the inclusion of CHWs in team meetings and rounds where appropriate.

Moving forward, it is crucial to expand awareness of the benefit CHWs can bring to patients (2, 3). Education about funding mechanisms and role definition for CHWs were noted as particular concerns of clinicians, thus standardized education for epilepsy center teams about these facets of CHW integration in particular is needed for clinicians and other staff at medical centers. Ensuring that clinicians on epilepsy center teams merit and support the role of a CHW as a healthcare professional able to serve as a liaison between vulnerable patients and their epilepsy center medical team, and as skilled workers who can provide patient education and help patients to navigate healthcare systems and related care transitions, as well as insurance and social service systems in order to meet patient needs and improve outcomes. The need to address key factors influencing care access and outcomes are well known in epilepsy, and the example of transportation highlights the significance of need; people with active epilepsy are greater than five times more likely than the general population to lack transportation to healthcare services (13). Further, paramount to achieving seizure control for people with epilepsy is recognizing that many struggle with access and adherence to anti-seizure medication treatment (37, 38). As evidenced by CHW roles in other chronic diseases (39), implementing CHW led standardized screening for anti-seizure medication adherence barriers and a prompt response to individual patient needs would bring imperative attention to this well-known challenge and could be an impactful epilepsy center strategy that decreases risk for seizure related complications (40).

In addition to awareness of the potential benefits, care teams and institutions must have awareness around an available work force of epilepsy trained CHWs to recruit and hire. Virtually accessible epilepsy-specific accredited CHW curriculum developed at Dartmouth (EK) has allowed for the ongoing provision of this training to partners nationwide since 2020 through collaboration with the Southern New Hampshire Area Health Education Center, the New England Public Health Training Center, the Rural CHW Network, and the National Community Health Worker Association. Continuing to build a skilled workforce of epilepsy-specific CHWs is necessary to address health disparities in people with epilepsy in epilepsy center environments, but also broadly in all settings where people with epilepsy receive care from health professionals.

Progress on the front of CHW integration in specialty clinical care settings, including at epilepsy centers, requires the development of strategies and practical avenues for sustainable funding. CHWs have mainly been funded under short term non-profit, state or federal grant programs designed for pilot projects and infrastructure development not for long term implementation of a CHW role. Next steps to partner collectively with epilepsy serving professional organizations (American Epilepsy Society) and state based professional CHW organizations and their licensing bodies to advocate with payors for a structured approach to integrating epilepsy training for CHWs that could lead to improving patient access to CHW services covered by medical insurance. Examining, healthcare reform-related alternative payment structures and internal funding by provider groups and institutions in anticipation of reduced costs and a return on CHW integration advancement are two potential avenues that must be explored for overcoming sustainable funding hurdles.

### 4.1 Limitations

This study has three limitations. First, findings are based on self-reported data which is potentially subject to recall and interviewer bias; this could present inaccuracies. Interviews were coded by multiple researchers not involved in the interviews to minimize potential interviewer bias. Second, this study focused solely on gaining clinician perspectives on the integration of CHWs onto epilepsy center care teams, from epilepsy center staff. We attempted to balance perspectives from different professional roles with the knowledge that there is a great paucity of epilepsy-specific CHWs integrated on teams currently. It is also possible that neurologists, epileptologists, nurses and social workers not affiliated with an accredited epilepsy center have different perceptions and experiences. However, bringing forward an initial understanding of traditional epilepsy center clinician perspectives not previously explored is valuable; without elucidating these perspectives, it is difficult to know how to integrate a CHW onto an epilepsy care team and what perceived barriers may exist. Future research integrating CHW and patient perspectives will allow for a more comprehensive understanding of the issues and help to balance clinician viewpoints. Third, these data were collected from clinicians at 5 epilepsy centers in New England and therefore may not be generalizable to other areas of the country. This geographic limitation could limit applicability of results to other regions or care settings, however, within our group of epilepsy centers, both urban and rural settings were included. Although we acknowledge the limited geography and focus on clinician perceptions, thematic saturation was reached when coding the interviews. Irrespective of clinic location (i.e., rural versus urban), themes were consistent. Challenges posed by epilepsy impact the spectrum of individuals diagnosed with this chronic and often unpredictable disease, irrespective of where they are located; clinicians are therefore also impacted broadly when facing resource and support needs for their epilepsy patient communities. As an example, while transportation may be more difficult for individuals with epilepsy living in rural areas in some circumstances, individuals living in urban areas also frequently experience the same hardships (11, 13).

## 5 Conclusion

The integration of CHWs into epilepsy center teams is a promising approach to bridge a divide between ideally available health care services, the quality of services received, and limiting the adverse impact of SDOH on people with epilepsy (11). Study findings have identified elements of an actionable model of CHW integration in epilepsy centers related to CHW roles and scope of practice, training, funding mechanisms, and team inclusion opportunities. This study makes evident that multidisciplinary clinician stakeholders are eager for new solutions to assist with filling patient SDOH care gaps and share a desire for replicable and sustainable models to integrate CHWs into epilepsy clinical care settings. Efforts to further expand our understanding of the perspectives of patients and CHWs are also required. Raising awareness around the availability of, and satisfaction with, accredited accessible virtual epilepsy-specific training programs for CHWs^2^ could facilitate interest in the feasibility of CHW integration on epilepsy care teams (41). Investment in CHW integration at epilepsy centers (42) may allow for improved working conditions for clinicians and better health outcomes for patients.

## Data Availability

The raw data supporting the conclusions of this article will be made available by the authors, without undue reservation.

## Footnotes

1
https://cdn.auckland.ac.nz/assets/psych/about/our-research/documents/Answers%20to%20frequently%20asked%20questions%20about%20thematic%20analysis%20April%202019.pdf

2 https://managingepilepsywell.org/community-health-worker; https://www.dartmouth-hitchcock.org/hobscotch-institute/community-training

## References

[1] American Public Health Association. Community health workers. (2024). Available online at: https://www.apha.org/apha-communities/member-sections/community-health-workers. 10.2190/NS.20.3.l 20943481

[2] Balcazar H Rosenthal EL Brownstein JN Rush CH Matos S Hernandez L. Community health workers can be a public health force for change in the United States: three actions for a new paradigm. Am J Public Health. (2011) 101:2199–203. doi: 10.2105/AJPH.2011.300386, PMID: 22021280 PMC3222447

[3] Rosenthal EL Brownstein JN Rush CH Hirsch GR Willaert AM Scott JR . Community health workers: part of the solution. Health Aff. (2010) 29:1338–42. doi: 10.1377/hlthaff.2010.0081, PMID: 20606185

[4] Martinez J Ro M Villa NW Powell W Knickman JR. Transforming the delivery of care in the post-health reform era: what role will community health workers play? Am J Public Health. (2011) 101:e1–5. doi: 10.2105/AJPH.2011.300335, PMID: 22021289 PMC3222444

[5] Otero-Sabogal R Arretz D Siebold S Hallen E Lee R Ketchel A . Physician-community health worker partnering to support diabetes self-management in primary care. Qual Prim Care. (2010) 18:363–72. PMID: 21294977. PMID: 21294977

[6] Marcotte L Dugdale D. Prevention as a population health strategy. Prim Care Clin Office Pract. (2019) 46:493–503. doi: 10.1016/j.pop.2019.07.004, PMID: 31655746

[7] Collinsworth A Vulimiri M Snead C Walton J. Community health workers in primary care practice: redesigning health care delivery systems to extend and improve diabetes care in underserved populations. Health Promot Pract. (2014) 15:51S–61S. doi: 10.1177/1524839914539961, PMID: 25359249

[8] Pinto D Carroll-Scott A Christmas T Heidig M Turchi R. Community health workers: improving population health through integration into healthcare systems. Curr Opin Pediatr. (2020) 32:674–82. doi: 10.1097/MOP.0000000000000940, PMID: 32889962

[9] George R Gunn R Wiggins N Rowland R Davis MM Maes K . Early lessons and strategies from statewide efforts to integrate community health workers into Medicaid. J Health Care Poor Underserved. (2020) 31:845–58. doi: 10.1353/hpu.2020.0064, PMID: 33410811 PMC7791094

[10] Avetisyan R Cabral H Montouris G Jarrett K Shapiro GD Berlowitz DR . Evaluating racial/ethnic variations in outpatient epilepsy care. Epilepsy Behav. (2013) 27:95–101. doi: 10.1016/j.yebeh.2012.12.020, PMID: 23399943

[11] Szaflarski M Wolfe JD Tobias JGS Mohamed I Szaflarski JP. Poverty, insurance, and region as predictors of epilepsy treatment among US adults. Epilepsy Behav. (2020) 107:107050. doi: 10.1016/j.yebeh.2020.107050, PMID: 32294594 PMC7242147

[12] Bensken WP Navale SM Andrew AS Jobst BC Sajatovic M Koroukian SM. Markers of quality care for newly diagnosed people with epilepsy on Medicaid. Med Care. (2021) 59:588–96. doi: 10.1097/MLR.0000000000001541, PMID: 33797505 PMC8187271

[13] Thurman DJ Kobau R Luo YH Helmers SL Zack MM. Health-care access among adults with epilepsy: the U.S. National Health Interview Survey, 2010 and 2013. Epilepsy Behav. (2016) 55:184–8. doi: 10.1016/j.yebeh.2015.10.028, PMID: 26627980 PMC5317396

[14] Swider SM. Outcome effectiveness of community health workers: an integrative literature review. Public Health Nurs. (2002) 19:11–20. doi: 10.1046/j.1525-1446.2002.19003.x, PMID: 11841678

[15] Spencer MS Rosland AM Kieffer EC Sinco BR Valerio M Palmisano G . Effectiveness of a community health worker intervention among African American and Latino adults with type 2 diabetes: a randomized controlled trial. Am J Public Health. (2011) 101:2253–60. doi: 10.2105/AJPH.2010.300106, PMID: 21680932 PMC3222418

[16] Norris SL Chowdhury FM Van Le K Horsley T Brownstein JN Zhang X . Effectiveness of community health workers in the care of persons with diabetes. Diabet Med. (2006) 23:544–56. doi: 10.1111/j.1464-5491.2006.01845.x 16681564

[17] Hood CM Gennuso KP Swain GR Catlin BB. County health rankings: relationships between determinant factors and health outcomes. Am J Prev Med. (2016) 50:129–35. doi: 10.1016/j.amepre.2015.08.024, PMID: 26526164

[18] Thornton RL Glover CM Cené CW Glik DC Henderson JA Williams DR. Evaluating strategies for reducing health disparities by addressing the social determinants of health. Health Aff. (2016) 35:1416–23. doi: 10.1377/hlthaff.2015.1357, PMID: 27503966 PMC5524193

[19] Viswanathan M Kraschnewski J Nishikawa B Morgan LC Thieda P Honeycutt A . Outcomes of community health worker interventions. Evid Rep Technol Assess. (2009) 181:1-144, A1-2, B1-14. PMC4781407 20804230

[20] Guide TC. Diabetes prevention: interventions engaging community health workers. (2022). Available online at: https://www.thecommunityguide.org/topics/diabetes.html.

[21] Guide TC. Cardiovascular disease: interventions engaging community health workers: guide to community preventive services. (2020). Available online at: https://www.thecommunityguide.org/findings/heart-disease-stroke-prevention-interventions-engaging-community-health-workers.html.

[22] Kangovi S Mitra N Grande D Long JA Asch DA. Evidence-based community health worker program addresses unmet social needs and generates positive return on investment. Health Aff. (2020) 39:207–13. doi: 10.1377/hlthaff.2019.00981, PMID: 32011942 PMC8564553

[23] Kiriakopoulos E Murray A Jobst B, eds. Implementing social determinants of health screening in the epilepsy clinic. American Epilepsy Society Meeting. (2022). Nashville, TN: American Epilepsy Society.

[24] Braun V Clarke V. What can "thematic analysis" offer health and wellbeing researchers? Int J Qual Stud Health Well-being. (2014) 9:26152. doi: 10.3402/qhw.v9.26152, PMID: 25326092 PMC4201665

[25] Braun V Clarke V. Reflecting on reflexive thematic analysis. Qual Res Sport, Exerc Health. (2019) 11:589–97. doi: 10.1080/2159676X.2019.1628806

[26] Braun V Clarke V. One size fits all? What counts as quality practice in (reflexive) thematic analysis? Qual Res Psychol. (2021) 18:328–52. doi: 10.1080/14780887.2020.1769238

[27] Clarke V Braun V. Successful qualitative research: a practical guide for beginners. QMiP Bulletin. (2013)

[28] Clarke V Braun V. Thematic analysis. Encyclopedia of critical psychology. (2014). New York: Springer. 32014: 1947–1952.

[29] O'Brien BC Harris IB Beckman TJ Reed DA Cook DA. Standards for reporting qualitative research: a synthesis of recommendations. Acad Med. (2014) 89:1245–51. doi: 10.1097/ACM.0000000000000388, PMID: 24979285

[30] Ponterotto J. Brief note on the origins, evolution, and meaning of the qualitative research concept "thick description". Qual Rep. (2006) 11:538–49. doi: 10.46743/2160-3715/2006.1666, PMID: 39386316

[31] Findley S Matos S Hicks A Chang J Reich D. Community health worker integration into the health care team accomplishes the triple aim in a patient-centered medical home: a Bronx tale. J Ambul Care Manage. (2014) 37:82–91. doi: 10.1097/JAC.0000000000000011 24309397

[32] Johnson SL Gunn VL. Community health workers as a component of the health care team. Pediatr Clin N Am. (2015) 62:1313–28. doi: 10.1016/j.pcl.2015.06.004, PMID: 26318954

[33] Applegate WB Ouslander JG Kuchel GA. Implementing “patient-centered care”: a revolutionary change in health care delivery. J Am Geriatr Soc. (2018) 66:1863–5. doi: 10.1111/jgs.15536, PMID: 30281789

[34] Wagle N Campbell C Di Caputa P Kocher B. A clinical pathway to well-being: putting patient priorities at the center of care. Am J Accountable Care. (2023) 11:37–40. doi: 10.37765/ajac.2023.89437

[35] McCarville EE Martin MA Pratap PL Pinsker E Seweryn SM Peters KE. Understanding the relationship between care team perceptions about CHWs and CHW integration within a US health system, a qualitative descriptive multiple embedded case study. BMC Health Serv Res. (2022) 22:1587. doi: 10.1186/s12913-022-08723-7, PMID: 36575412 PMC9793519

[36] Wagner EH Flinter M Hsu C Cromp D Austin BT Etz R . Effective team-based primary care: observations from innovative practices. BMC Fam Pract. (2017) 18:13. doi: 10.1186/s12875-017-0590-8, PMID: 28148227 PMC5289007

[37] Gutierrez-Colina AM Smith AW Mara CA Modi AC. Adherence barriers in pediatric epilepsy: from toddlers to young adults. Epilepsy Behav. (2018) 80:229–34. doi: 10.1016/j.yebeh.2018.01.031, PMID: 29433948 PMC6816538

[38] O’Rourke G O’Brien JJ. Identifying the barriers to antiepileptic drug adherence among adults with epilepsy. Seizure. (2017) 45:160–8. doi: 10.1016/j.seizure.2016.12.006, PMID: 28063375

[39] Jacob V Chattopadhyay SK Hopkins DP Reynolds JA Xiong KZ Jones CD . Economics of community health workers for chronic disease: findings from community guide systematic reviews. Am J Prev Med. (2019) 56:e95–e106. doi: 10.1016/j.amepre.2018.10.009, PMID: 30777167 PMC6501565

[40] Donahue MA Akram H Brooks JD Modi AC Veach J Kukla A . Barriers to medication adherence in people living with epilepsy. Neurol Clin Pract. (2025) 15:e200403. doi: 10.1212/CPJ.0000000000200403, PMID: 39610394 PMC11604104

[41] Kiriakopoulos ET Dawson T Schmidt S Jobst B. Pilot implementation of virtual epilepsy and self-management training for community health workers. Proceedings of the American Epilepsy Society Meeting. AES: 2021, Dec 3–7. Chicago (IL): American Epilepsy Society. (2021).

[42] Kiriakopoulos E Murray A Jobst B. Implementing social determinants of health screening in the epilepsy clinic. Proceedings of the American Epilepsy Society Meeting. AES: 2022, Dec 2–6. Chicago (IL): American Epilepsy Society. (2022).

