# Supplementary material for: A qualitative analysis of clinician perspectives on community health worker integration at epilepsy centers
Source: Front Neurol. 2025 Apr 9;16:1560077. doi: 10.3389/fneur.2025.1560077 (PMC12014466; doi:10.3389/fneur.2025.1560077)
Supplement: Supplementary file 1 [file Supplementary_file_1.pdf]

APPENDIX A: CLINICIAN INTERVIEW GUIDE

MAIN QUESTION	ADDITIONAL QUESTIONS	CLARIFYING QUESTIONS
**Prior Knowledge of CHWs**<br>A good place to start would be to ask if you could share, what knowledge do you have around CHWs?	Do you have any firsthand experience interacting with CHWs?<br><br>Have you heard from colleagues, or read about the utility of CHW as integrated members of a clinical team?	Can you expand a little on this?<br><br>Can you tell me anything else?<br><br>Can you give me some examples?
**Recruitment and Selection**<br>***If no CHW on team***<br>Do you have knowledge about how to recruit and select a CHW?<br><br>***If CHW already present***<br>How did you recruit and select your CHW?	***If no CHW on team***<br>Are there supports at your medical center to help you with the process of recruiting and selecting a CHW?<br><br>***If CHW already present***<br>Are there any changes you would make to your recruitment and selection process?	Can you expand a little on this?<br><br>Can you tell me anything else?<br><br>Can you give me some examples?
**Roles and Responsibilities of CHW on an Epilepsy team**<br>***If no CHW on team***<br>Do you feel you have a good idea of the role a CHW could play in a clinical epilepsy center setting?<br><br>***If CHW already present***<br>Can you share the role of the CHW on your team and what responsibilities are assigned to them?	***If no CHW on team***<br>If you had a CHW on your team, what specific responsibilities would you feel appropriate and comfortable to assign to the CHW?<br><br>***If CHW already present***<br>How well do you think they are able to fulfill their role and the assigned responsibilities?	Can you expand a little on this?<br><br>Can you tell me anything else?<br><br>Can you give me some examples?
**Training and Supervision of CHW**<br>***If no CHW on team***<br>What level of training would you want a CHW to have in order to participate as a member of the team caring for patients?	***If no CHW on team***<br>Are you aware of training programs specific to CHWs?<br><br>Who would be best on your team to supervise a CHW? Why?	Can you expand a little on this?<br><br>Can you tell me anything else?<br><br>Can you give me some examples?

MAIN QUESTION	ADDITIONAL QUESTIONS	CLARIFYING QUESTIONS
**Training and Supervision of CHW**<br>***If CHW already present***<br>Does the CHW on your team have training specific to the role of a CHW or epilepsy?<br>Who supervises your CHW? And is that model working?	***If CHW already present***<br>Are you aware of the CDC Epilepsy and Self-Management training available for CHWs?<br><br>Do you feel like the level of supervision received is adequate?	Can you expand a little on this?<br><br>Can you tell me anything else?<br><br>Can you give me some examples?
**Funding to Support and Sustain a CHW**<br><br>***If no CHW on team***<br>Is there a funding mechanism you are aware of to allow you to integrate a CHW onto your team?<br><br>***If CHW already present***<br>How do you support the salary of your CHW?	What concerns do you have around salary support and sustainability of the CHW position?	Can you expand a little on this?<br><br>Can you tell me anything else?<br><br>Can you give me some examples?
**SDOH Care Gaps at epilepsy center**<br><br>Do you feel in your care of patients that there is an emphasis on addressing SDOH for your patient population?<br><br>How does that present? And do you gather data on meeting SDOH for patient needs currently?	Do you think you are meeting the needs of your patients when it comes to SDOH?<br><br>How much of an impact is this having on your ability to provide care and ultimately their health?	Can you expand a little on this?<br><br>Can you tell me anything else?<br><br>Can you give me some examples?
**Confidence in the abilities of a non-medical provider to work with your patients and better their health**<br><br>***If no CHW on team***<br>What confidence do you have that a nonmedical provider could integrate well onto your team in a way that benefits the team, and the patient experience?<br><br>***If CHW already present***<br>What was your confidence level in working with the CHW on your team?	What reservations did/or do you have about the CHW role?<br><br>What potential did/do you see in having a CHW on your team long term?	Can you expand a little on this?<br><br>Can you tell me anything else?<br><br>Can you give me some examples?
