# Supplementary material for: A qualitative analysis of clinician perspectives on community health worker integration at epilepsy centers
Source: Front Neurol. 2025 Apr 9;16:1560077. doi: 10.3389/fneur.2025.1560077 (PMC12014466; doi:10.3389/fneur.2025.1560077)
Supplement: Supplementary file 2 [file Supplementary_file_2.pdf]

Supplementary Material – Appendix B. Clinician Interview Guide

Article Title: A qualitative analysis of clinician perspectives on community health worker integration at epilepsy centers

Correspondence: Elaine T. Kiriakopoulos, MD, MPH, MSc
elaine.t.kiriakopoulos@dartmouth.edu

APPENDIX B: FINAL CODEBOOK

(Please indicate number and letter, 1a, 2c etc.)<br>**CODEBOOK for**<br>**CLINICIAN INTERVIEWS**		
Note: Codes listed represent options available to researchers coding the qualitative interviews. Not all codes were represented in final data.		
	**CODE**	**DESCRIPTION**
**1**	*Prior Knowledge of Community Health Worker (CHW)*<br>**a. No prior knowledge**<br>**b. Has heard of CHWs but unsure what defines a CHW**<br>**c. Worked with them directly in past and experience was positive**<br>**d. Worked with them directly in past and experience was negative**<br>**e. Worked with them only peripherally (as an observer of their work) in past**<br>**f. Aware other departments at medical center utilize CHWs**<br>**g. Currently work with CHW**	Interviewee expresses their personal knowledge level for what a CHW is and any past or present experience with a CHW; shares knowledge of CHW at their medical center.
**2**	*CHW Recruitment and Selection*<br>**a. HR at medical center**<br>**b. Person being interviewed feels they could fulfill these tasks**<br>**c. Team member identified who could be a recruiter or selector**<br>**d. No idea how to recruit or select**<br>**e. Expresses characteristics for selection they think would be important**	Interviewee expresses routes for recruiting a CHW or the ability to select a CHW; describes systems/people in place or not in place to help with this at their medical center.<br><br>Interviewee shares their awareness level for what characteristics important for selection.
**3**	*CHW Roles and Responsibilities*<br>**a. Differentiating clinic work and community work**<br>**b. Home visits**<br>**c. Comparison to social work role**<br>**d. Lessening workload for nursing**<br>**e. Addressing transportation needs**<br>**f. Addressing financial needs**<br>**g. Addressing insurance needs**<br>**h. Addressing housing needs**	Interviewee describes potential roles for CHW on team, or responsibilities they feel could be ascribed to a CHW.<br><br>Interviewee provides specific examples of responsibilities, or impact their role/responsibilities would have on other team members.

	i. **Addressing food insecurity needs**<br>j. **Addressing medication access**<br>k. **Educating on medication adherence**<br>l. **Connecting to community resources**<br>m. **Liaison between patient and med team**<br>n. **Provide patient education (epilepsy, seizure first aid)**<br>o. **Connecting to counselling/mental health services and providers**<br>p. **Connect patients to self-management programs**<br>q. **Assist patients with forms related to their health and well being**<br>r. **Assisting with employment forms/programs**	Interviewee describes how a CHW might assist nursing team workload around patient education, transportation, forms, medication adherence (helping with acquiring medication, forms for patient assistance, mail delivery of medication, etc.)
**4**	*CHW Training and Supervision*<br>a. **Formal education (college education)**<br>b. **High school education/GED**<br>c. **Informal training programs at medical center**<br>d. **State or other certification program for CHWs**<br>e. **Knowledge of epilepsy specific CHW training (Centers for Disease Control/Dartmouth)**<br>f. **Social work as supervisor**<br>g. **Nursing as supervisor**<br>h. **Doctor as supervisor**<br>i. **Supervisor of CHWs at medical center**	Interviewee describes the level of training/certification they feel would be best for a CHW who is being integrated on their team; shares views on who might be best to provided day to day supervision.<br><br>Interviewee describes any knowledge they have about training and supervision of a CHW.
**5**	*CHW Funding Mechanisms*<br>a. **Medical center support is present or could be**<br>b. **External funding avenues (grants, local or federal/state programs)**<br>c. **Insurance reimbursement**<br>d. **No idea where to look for funding for a CHW**<br>e. **Unlikely department could fund CHW position**<br>f. **Would be sustainable if department decision made to integrate a CHW**<br>g. **A CHW role would not be sustainable**	Interview shares potential known sources to bring financial support for the role of a CHW; or shares they are uncertain how a CHW could be funded.<br><br>Interviewee expresses thoughts on limits to funding at their center or centers in general; describes hurdles or support that may exist.
**6**	*Epilepsy Team/Care Gaps*<br>a. **Addressing social determinants of health (SDOH)**<br>b. **Ability to track SDOH needs met**<br>c. **Unsure of SDOH tracking/needs being met**<br>d. **Social work addressing SDOH**<br>e. **Nursing team addressing SDOH**<br>f. **SDOH high need variables for PWE**<br>g. **Lack of staffing to address SDOH**<br>h. **Lack of funding to support staff time for SDOH work**<br>i. **A CHW would be seen as beneficial/could work with other team members to address SDOH needs**	Interviewee shares current epilepsy team gaps in care and how they are/or could be addressing.<br><br>Interviewee shares if they are working actively to address SDOH and if they are tracking SDOH and positive/negative outcomes.<br><br>Interviewee indicates areas of high need (i.e., transportation).<br><br>Gaps related to staffing or funding

7	*Expectations and Trust*<br>**a. Believe there is/would be benefit to patients**<br>**b. CHW could be trusted with private and sensitive patient information**<br>**c. Patients view CHW as positive**<br>**d. Patient only wants to see a medical provider**<br>**e. No concern**<br>**f. Some concerns**	Interviewee shares what they would expect from a CHW and how trust may factor in to integration on team.<br><br>Interviewee shares their perception on benefits of a CHW on the team.<br><br>Interviewee shares their perception regarding patient trust or expectations.<br><br>Interview shares concerns about the CHW role or problems that might arise.
8	*Culture of Collaboration*<br>**a. Open to role of CHW**<br>**b. Believe CHW would be well received by epilepsy team members**<br>c. **Good team mechanics exist to integrate a CHW**<br>**d. Resistance to CHW integration**	Interview discusses thoughts of CHW integration into the epilepsy center team and potential barriers and facilitators.<br>Interviewee shares methods for shared communication with team members; involvement in existing team conferences/rounds; EMR participation.
9	*Good Quote*	Exemplar comments that reflect a general sentiment/recurring idea from pool of interviews; or displaying an innovative concept or perception.
